# Associations between app usage and behaviour change in a m-health intervention to improve physical activity and sleep health in adults: secondary analyses from two randomised controlled trials

**DOI:** 10.1186/s44167-022-00013-1

**Published:** 2023-02-02

**Authors:** Leah L. Murphy, Ben J. Dascombe, Beatrice Murawski, Anna T. Rayward, Wendy J. Brown, Ronald C. Plotnikoff, Corneel Vandelanotte, Elizabeth G. Holliday, Mitch J. Duncan

**Affiliations:** 1 School of Medicine and Public Health, Faculty of Health and Medicine, College of Health, Medicine, and Wellbeing, University of Newcastle, University Drive, Callaghan, 2308 NSW Australia; 2 Active Living Research Program, Hunter Medical Research Institute, New Lambton Heights, NSW 2305 Australia; 3 Applied Sport Science and Exercise Testing Laboratory, School of Environmental and Life Sciences, University of Newcastle, Ourimbah, NSW 2258 Australia; 4 School of Education, University of Newcastle, Callaghan, NSW 2308 Australia; 5 School of Human Movement and Nutrition Studies, The University of Queensland, Brisbane, QLD 4072 Australia; 6 Faculty of Health Sciences and Medicine, Bond University, Gold Coast, QLD 4226 Australia; 7 School of Health, Medical and Applied Sciences, Appleton Institute, Physical Activity Research Group, Central Queensland University, Rockhampton, QLD 4701 Australia

**Keywords:** eHealth, Usage, Engagement, Attrition, Dose-response

## Abstract

**Background:**

To examine associations between user engagement and activity-sleep patterns in a 12-week m-health behavioural intervention targeting physical activity and sleep.

**Methods:**

This secondary analysis used data pooled from two Randomised Control Trials (RCT, [Synergy and Refresh]) that aimed to improve physical activity and sleep (PAS) among physically inactive adults with poor sleep. Both RCTs include a PAS intervention group (n = 190 [Synergy n = 80; Refresh n = 110]) and a wait list Control (CON n = 135 [Synergy n = 80; Refresh n = 55]). The PAS groups received a pedometer and accessed a smartphone/tablet “app” with behaviour change strategies, and email/SMS support. Activity-sleep patterns were quantified using the activity-sleep behaviour index (ASI) based on self-report measures. Intervention usage was quantified as a composite score of the frequency, intensity and duration of app usage during intervention (range: 0–30). Assessments were conducted at baseline, 3 and 6 months. Relationships between usage and ASI were examined using generalised linear models. Differences in ASI between the control group and intervention usage groups (Low [0–10.0], Mid [10.1–20.0], High [20.1–30.0]) were examined using generalised linear mixed models adjusted for baseline values of the outcome. Trial Registration: ACTRN12617000376347; ACTRN12617000680369.

**Results:**

During the 3-month intervention, the mean (± sd) usage score was 18.9 ± 9.5. At 3 months (regression coefficient [95%CI]: 0.45 [0.22, 0.68]) and 6 months (0.48 [0.22, 0.74]) there was a weak association between usage score and ASI in the intervention group. At 3 months, ASI scores in the Mid (Mean [95%CI] = 57.51 [53.99, 61.04]) and High (60.09 [57.52, 62.67]) usage groups were significantly higher (better) than the control group (51.91 [49.58, 54.24]), but not the Low usage group (47.49 [41.87, 53.12]). Only differences between the high usage and control group remained at 6 months.

**Conclusion:**

These findings suggests that while higher intervention usage is associated with improvements in behaviour, the weak magnitude of this association suggests that other factors are also likely to influence behaviour change in m-health interventions.

*Trial registration number:* ACTRN12617000376347; ACTRN12617000680369.

**Supplementary Information:**

The online version contains supplementary material available at 10.1186/s44167-022-00013-1.

## Introduction

Delivering behaviour change interventions through electronic health (e-Health) and mobile health (m-Health) creates the opportunity to deliver cost-effective wide-reaching interventions. Both e- and m-Health interventions have demonstrated effectiveness for improving lifestyle behaviours including physical activity [1], sleep [2], alcohol consumption and chronic disease management [3, 4]. Approximately 30% of adults are both physically inactive and have poor sleep health [5, 6] and may benefit from interventions to improve these behaviours. However, there are few interventions that have targeted improvements in both physical activity and sleep [7, 8]. Consequently, relatively little is known about how participants use and engage with digital physical activity and sleep interventions. Characterising engagement with digital interventions that target multiple lifestyle behaviours is important given the number of adults who engage in multiple higher risk behaviours [9] and the need for interventions to address multiple lifestyle behaviours concurrently.

Understanding how participants use and engage with digital interventions is important, as while it is typically assumed that greater usage is associated with greater behaviour change, the magnitude of this relationship appears to be weak [10, 11]. Furthermore, it is consistently reported that usage declines throughout the intervention period [12, 13]. Additionally, there are inconsistencies between studies regarding how usage is conceptualised and measured, which limits comparisons between studies [11, 14–17]. A systematic review reported that a greater subjective user experience of the intervention, greater number of activities completed and more frequent logins are consistently associated with greater physical activity, but that time on the website was not associated with physical activity [11]. This suggests the usage-behaviour change relationship may differ depending on the usage metric examined and that single usage metrics may not adequately characterise how participants use and engage with the intervention. To overcome this, Short and colleagues [15] proposed a composite measure of usage that captures the frequency (i.e., number of self-monitoring entries or logins), intensity (i.e., number of intervention features used), duration, and type (i.e., reflective, didactic, or active) of usage. Such composite measures may be more useful in understanding the usage-behaviour change relationship [14–16], although few studies have applied such multidimensional measures. The overall aim of this study was to examine how user engagement with a m-health app is associated with behaviour change during two randomised controlled trials (RCT) of a m-health intervention targeting improvements in physical activity and sleep health in physically inactive adults with poor sleep. Two specific objectives were to: (1) examine the relationship between user engagement and a composite score of overall physical activity and sleep health (Activity-Sleep Index [ASI]) within the intervention group and, (2) compare differences in overall physical activity and sleep health in the control group and in different levels of app usage (Low, Mid, High) in the intervention group.

## Methods

### Study design

This study uses data pooled from two separate RCTs of the same m-health intervention, which was designed to improve physical activity and sleep health behaviours in physically inactive adults with poor sleep quality [7, 8]. Details of the study rationale, methods and main outcomes of each trial, and intervention effects on the ASI are available elsewhere [7, 8, 18–20]. Similarities between the trials in terms of the behaviours assessed, intervention and control groups, assessment methods and outcomes assessed allowed data from the control and physical activity and sleep health intervention groups to be pooled as described previously [20]. Eligible participants were those aged 18–55 years (Synergy Study [7, 18]) or 45–65 years (Refresh Study [8, 19]), who lived in Australia, reported < 90 min of moderate to vigorous intensity physical activity (MVPA) in the last week and rated their sleep quality as fairly bad or very bad. Exclusion criteria included being employed in shift-work, diagnosed sleep disorder, and current use of a device to track activity or sleep (see Additional file 1: Figs. S1, S2).

Both studies primarily recruited participants using social media advertising. The Synergy study aimed to compare the efficacy of a combined physical activity and sleep health intervention with a wait-list control. Participants (n = 160; mean age: 41.5 (SD = 9.9); 80% female) were recruited between June–August 2017 and the study was conducted between June 2017 and February 2018. The Refresh study aimed to compare the efficacy of a combined physical activity and sleep health intervention with a sleep health-only intervention and a wait-list control [19, 21]. Participants (n = 275; mean age: 52.0 (SD = 6.9); 83% female) were recruited between May–September 2017 and the study was conducted between June 2017 and March 2018. The combined physical activity and sleep health intervention in both trials was the same in terms of mode of delivery, theoretical basis, educational content, and behaviour change techniques used. The sleep health-only intervention arm (n = 110) in Refresh was omitted from the current study as participants in that group did not receive any physical activity intervention content. The active phase of the intervention in both studies ceased at the 3-month point. Both studies conducted online assessments at baseline, 3 months and 6 months and were prospectively registered with the Australian and New Zealand Clinical Trials Registry as well as received ethical approval (H-2016-0181, H-2016-0267) at the University of Newcastle. Participants in both trials provided informed consent. Each study used computer generated permuted block randomisation to develop the randomisation sequence, with group allocation concealed in sequentially numbered envelopes. Participants were not blinded to group allocation given the nature of the interventions.

### Study groups

The physical activity and sleep (PAS) intervention group (n = 190 [Synergy: n = 80 + Refresh: n = 110]) received access to a specifically designed mobile application “Balanced” that comprised educational resources, personal goals, self-monitoring logs (manual data entry), and feedback in relation to personal goals, all relative to a range of physical activity and sleep health components (i.e., activity minutes, step count, resistance training, bedtime, sleep wake timing and sleep quality). Details of the intervention are provided in Additional file 1: Tables S1 and Fig. S3. Prior to commencement, the intervention group participants were mailed a printed participant handbook with guidance on how to use the app, and a pedometer. Participants also received weekly reports and short message service (SMS) prompts to limit disengagement. Participants used the app for goal setting and action planning to increase their physical activity (i.e., MVPA, step counts and resistance training) and to improve their sleep quality and sleep behaviours (stabilising bed/wake times, sleep hygiene behaviours and stress management (e.g., progressive muscle relaxation, deep breathing exercises, mindfulness) [20]. All intervention components were delivered either through the application, email or SMS, and the messaging component ceased at three months. All self-monitoring data entered into the application were recorded in the application database, including the associated timestamp of entry. The waitlist-control group did not have any access to the application or other intervention materials prior to the 6-month assessment. However, they were offered full access to the intervention including the “Balanced” application after completing the final 6-month assessment.

### Measures

Sociodemographic variables such as age, gender, education and chronic disease status were assessed at baseline and primary and secondary outcomes were measured at baseline, three and six months [7, 8]. Primary and secondary measures of the original trials included minutes of MVPA [22], the frequency of resistance training [18, 19], sleep quality [23] and insomnia symptoms [24].

### Activity sleep index

The overall pattern of physical activity and sleep was quantified using the activity-sleep index (ASI), which is a 12-item instrument described elsewhere [20]. It is designed to assess overall healthy patterns of physical activity and sleep health based on the frequency, duration, type and intensity of physical activity, the duration of sitting time and the duration, timing, quality and satisfaction of sleep. The specific items, responses, and scoring for the ASI are provided in Additional file 1: Table S2. The items are briefly summarised here:

1. Frequency–*MVPA* (Number of sessions of MVPA/wk),
2. Frequency–*RT* (Number of days of resistance training/wk),
3. Intensity (Proportion of MVPA that was vigorous in intensity),
4. Type (Participation in no MVPA or resistance training, either MVPA or resistance training, or both),
5. Time (Duration of MVPA/wk),
6. Sitting (Duration of sitting time/wk).
7. Daytime alertness (Trouble staying awake during the day),
8. Sleep Quality (Overall sleep quality rating),
9. Sleep Timing (Midpoint of sleep between 02:00 am and 04:00 am),
10. Sleep Regularity (Variability in bed and wake times),
11. Sleep Efficiency (Sleep efficiency ([sleep duration/time in bed] × 100),
12. Sleep Duration (Meeting age-appropriate sleep duration guidelines).

To overcome the different metrics used to quantify each dimension, each dimension was rescaled to a zero to 10 scale, with higher scores reflecting lower risk behaviour. Each dimension is summed to create a score ranging from 0 to 120. The approach used to rescale the individual dimensions of the ASI was $Rescaled \, score=\left( \frac{X-{X}_{min}}{{X}_{Range} }\right)n$ where X is the observed value, X_*min*_ is the minimum observed value of the original variable, X_*Range*_ is the difference between the minimum and maximum of the observed values, and *n* is upper limit of the rescaled variable (e.g., n = 10) [20].

### Intervention usage

An overall usage score was created to capture intervention group participants’ frequency, intensity, and duration of using the “Balanced” intervention platform using data recorded in intervention database. There are no usage data for the wait-list control group as they did not have access to the app during the intervention period. Type of usage (i.e., reflective, gamified, altruistic, didactic, or active) was not examined as these data were not recorded in the intervention platform. All indicators were assessed over the initial 84-day (i.e., 3-month) intervention period to align with the ‘active’ component of the intervention. Each day, participants could self-report their: (1) minutes of MVPA, (2) resistance training, (3) daily step count, (4) bedtime, (5) wake time and, (6) sleep quality. The app was designed to promote daily self-monitoring of these metrics, however participants were free to self-monitor any number of these metrics on a given day. These measures were used to create measures of the frequency, intensity and duration of usage. Frequency was measured as the total number of self-monitoring entries made during the 3-month (84 days) intervention period, with a maximum of six entries per day (one entry per day for each of the self-monitoring entries made). Intensity was measured as the average number of self-monitoring entries made each day during the intervention. Duration was measured as the number of days until a participant succumbed to non-usage attrition, defined as the time they first stopped self-monitoring for at least 14 consecutive days [7, 21, 25, 26]. Due to the different metrics used to characterise each usage dimension (i.e., count of self-monitoring entries per day, number of days) each dimension was rescaled to a zero to 10 scale as follows rescaled score =$\left(\frac{X-{X}_{min}}{{X}_{range} }\right)n$ ; where X is the original score, X_min_ is the minimum of the observed variable, X_range_ is the range of the potential score and *n* is the upper limit of the rescaled score [20]. The rescaled dimensions were summed to create an overall usage score ranging from zero to thirty with higher values indicating greater usage.

### Data analysis

Descriptive statistics are presented for the sample at baseline in each intervention group, and also by intervention usage group. To examine the association between engagement and behaviour change, two separate analyses were conducted. The first analysis was limited to only the intervention group as no usage data were available for the control group. This analysis examined the relationship between the overall usage score and the ASI at follow-up, adjusted for the baseline value of the outcome, using a generalised linear model. The model included fixed effect for the continuous mean centred usage score, study (Refresh, Synergy), assessment (3 months, 6 months) and the interaction between usage score and assessment. The linearity of the relationship between continuous usage score and ASI in the intervention group was examined using residual plots and including a quadratic term for usage score in the analysis. The quadratic term was not statistically significant (p < 0.05) and the residuals plots did not indicate a non-linear relationship. To examine how varying amounts of usage in the intervention group were associated with behaviour change relative to the waitlist-control group, overall usage scores in the intervention group were categorised into a three-level group variable: low usage (0–10); mid usage (10.1–20.0); and high usage (20.1–30.0) and combined with the control group to create a four-level variable. This analysis examined between group differences (Control, Low usage, Mid usage, High usage) in the ASI adjusted for the baseline value of the outcome. The model included a fixed effect for study (Refresh, Synergy), assessment (3 months, 6 months), group (Control, Low usage, Mid usage, High usage), and the group by assessment interaction. Residual diagnostics were used to inform the choice of model and link. Analyses were conducted using Stata MP v17 and alpha was set at 0.05.

## Results

Participant flow throughout each trial is shown in Additional file 1: Figs. S1 and S2. A total of 325 participants completed the baseline survey, 275 (84.6%) completed the 3-month assessment, and 215 (66%) completed the 6-month assessment. Completers of the 3-month assessment were older (M = 47.41 [SD = 9.73]) and had higher levels of intervention usage (M = 20.73 [8.76]) than non-completers (Additional file 1: Table S3). The baseline sample consisted of 264 female and 61 male participants, and most were middle-aged and highly educated (Table 1). At baseline the average BMI and ASI were 28.15 kg/m^2^ (SD = 4.21) and 47.34 (SD = 10.91), respectively and both these variables were similar between intervention and control groups. At baseline the low usage group reported lower average ASI scores (M = 44.19 [SD = 14.11]), with a higher proportion reported higher income levels (≥$100,001/yr) relative to the Mid and High usage groups.

**Table 1:** Baseline descriptive characteristics of participants by study and intervention usage group

	Group		Intervention usage group			
	Control n = 135	Intervention n = 190	Low Usage n = 34	Mid Usage n = 60	High Usage n = 94	Total N = 325
	M (SD), n (%)	M (SD), n (%)	M (SD), n (%)	M (SD), n (%)	M (SD), n (%)	M (SD), n (%)
Age (years)	46.19 (10.39)	47.22 (9.71)	47.12 (9.37)	45.47 (10.53)	48.30 (9.29)	46.76 (10.02)
Education (years)	16.26 (2.90)	16.11 (2.77)	16.03 (3.05)	15.73 (2.99)	16.38 (2.53)	16.17 (2.82)
BMI	27.67 (4.09)	28.50 (4.27)	28.71 (4.48)	29.30 (4.33)	27.89 (4.10)	28.15 (4.21)
ASI Score	46.83 (10.03)	47.83 (11.56)	44.19 (14.11)	47.58 (10.54)	49.06 (10.90)	47.34 (10.91)
Sex						
Male	26 (19.26%)	35 (18.42%)	7 (20.59%)	12 (20.00%)	16 (17.02%)	61 (18.89%)
Female	109 (80.74%)	155 (81.58%)	27 (79.41%)	48 (80.00%)	78 (82.98%)	262 (81.11%)
Income/Yr						
≤ $30,000	32 (23.70%)	30 (15.79%)	2 (5.88%)	11 (18.33%)	17 (18.09%)	62 (19.20%)
$30,001–$50,000	14 (10.37%)	26 (13.68%)	3 (8.82%)	6 (10.00%)	17 (18.09%)	40 (12.38%)
$50,001–$70,000	26 (19.26%)	39 (20.53%)	8 (23.53%)	18 (30.00%)	13 (13.83%)	65 (20.12%)
$70,001–$100,000	25 (18.52%)	47 (24.74%)	7 (20.59%)	13 (21.67%)	26 (27.66%)	71 (21.98%)
≥ $100,001	30 (22.22%)	30 (15.79%)	10 (29.41%)	8 (13.33%)	11 (11.70%)	59 (18.27%)
Don’t know/no answer	8 (5.93%)	18 (9.47%)	4 (11.76%)	4 (6.67%)	10 (10.64%)	26 (8.05%)
Employment group						
Professional	85 (62.96%)	107 (56.32%)	21 (61.76%)	31 (51.67%)	55 (58.51%)	192 (59.44%)
White-collar	18 (13.33%)	37 (19.47%)	6 (17.65%)	16 (26.67%)	14 (14.89%)	54 (16.72%)
Blue-collar	5 (3.70%)	4 (2.11%)	0 (0.00%)	1 (1.67%)	3 (3.19%)	9 (2.79%)
Not working^a^	27 (20.00%)	42 (22.11%)	7 (20.59%)	12 (20.00%)	22 (23.40%)	68 (21.05%)

^a^Employment group not working includes retired, unemployed, home duties, looking for work, student, and other. Low, Mid and High usage defined as usage score of 0–10.0, 10.1–20.0 and 20.1–30.0, respectively

**Fig. 1:**
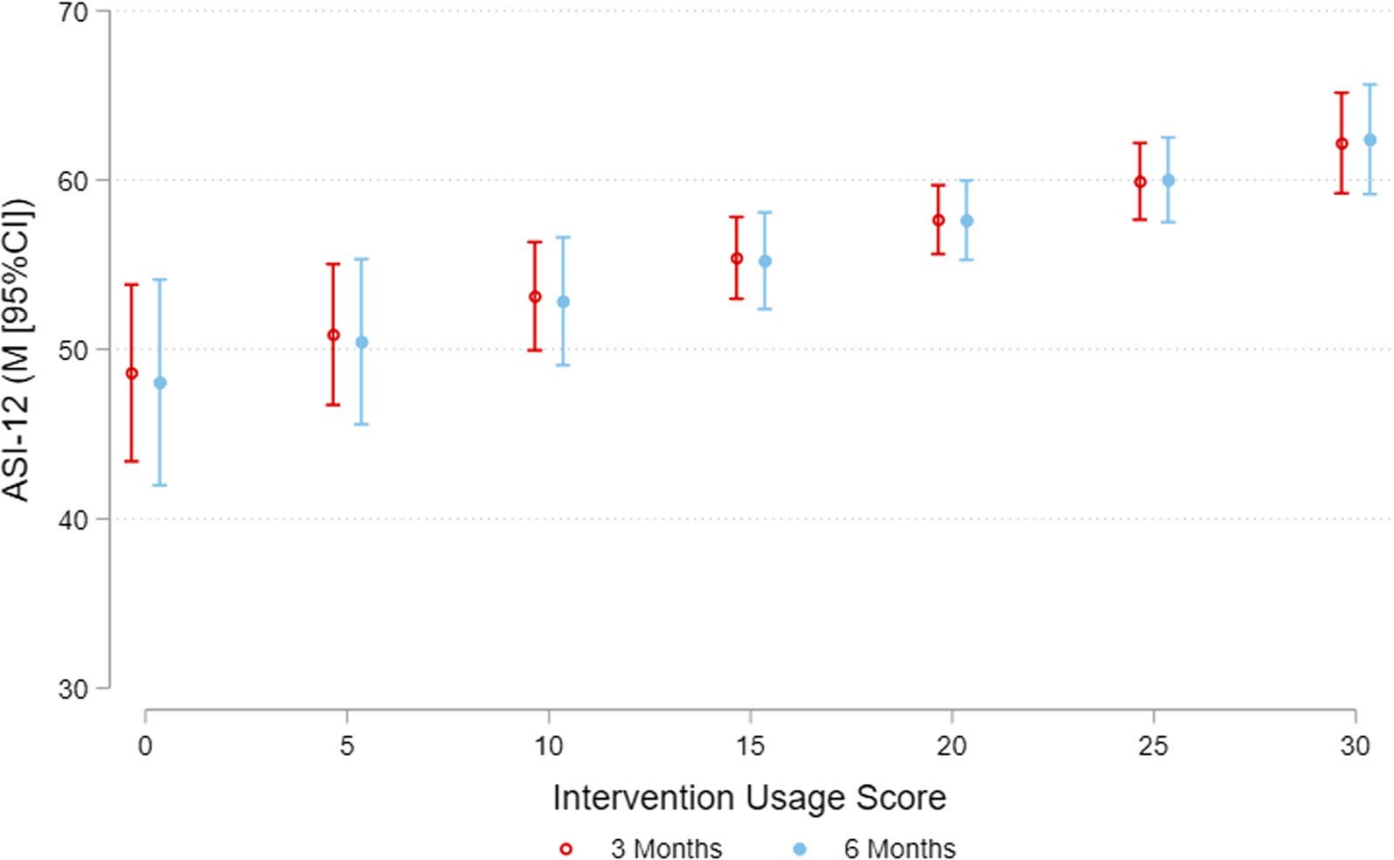
Baseline adjusted ASI-12 at 3 and 6 months by usage score in intervention group. Model only includes the pooled intervention group. Model adjusted for study (i.e., Synergy, Refresh), baseline ASI-12 score, and includes the mean centered usage score and its interaction with assessment. p-value for interaction between usage score and assessment is = 0.857 The association between usage score and ASI-12 at 3 months is = *Β*=, 95%CI: b = 0.45,95%CI = 0.22, 0.68 and at 6 months is *Β* = 0.48, 95%CI = 0.22, 0.74

**Fig. 2:**
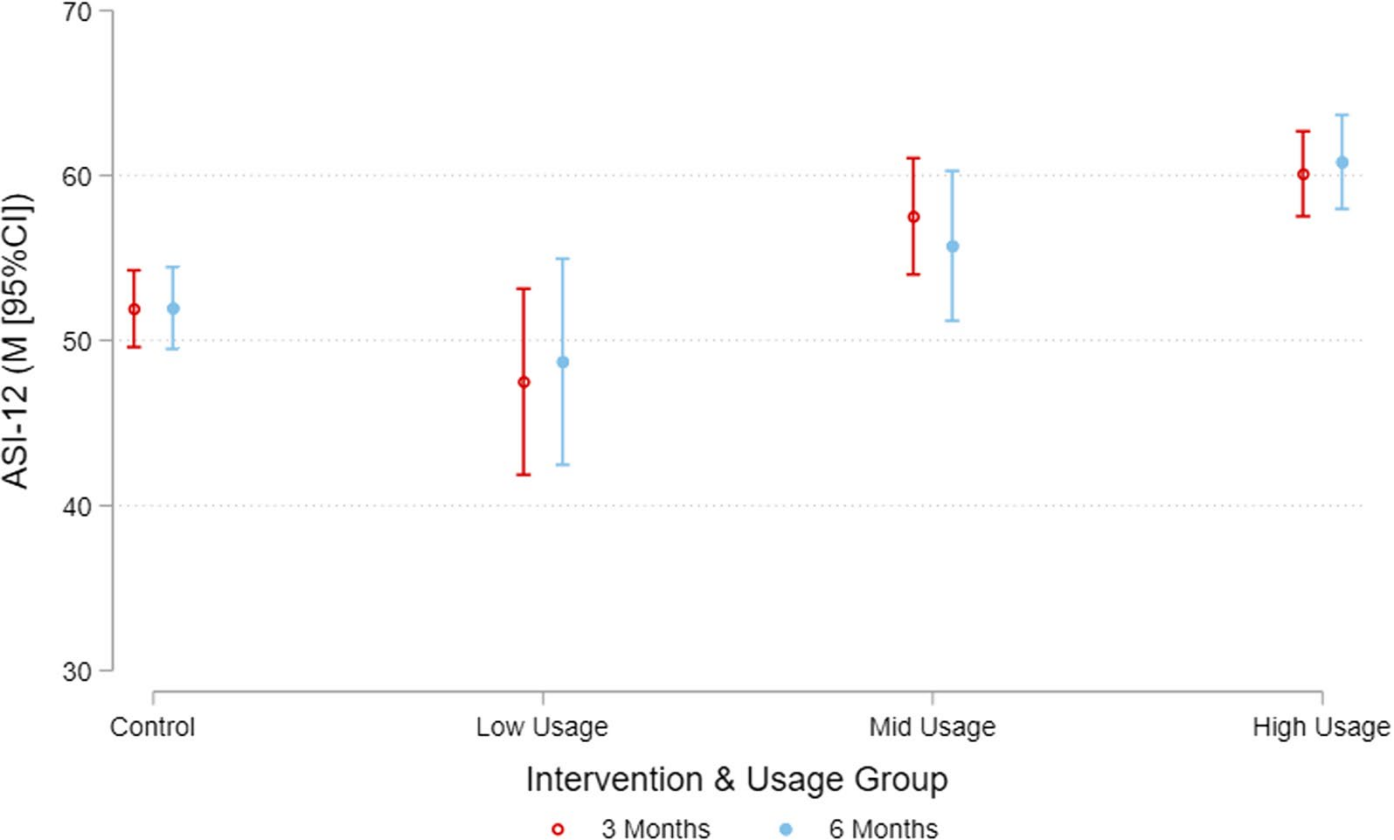
Baseline adjusted ASI-12 at 3 and 6 months by Intervention and Usage Group. Model adjusted for study (i.e., Synergy, Refresh), baseline ASI-12 score, fixed effects for group (Control, Low Usage, Mid Usage, High Usage), assessment (3 months, 6 months) and the group by assessment interaction

The average intervention usage score was 18.88 (SD = 9.54) out of 30, and the average usage scores in the Low, Mid and High usage groups were 3.49 (SD = 4.10), 14.78 (SD = 2.97), and 27.03 (SD = 3.00), respectively. In the intervention group, there was a weak association between intervention usage score and ASI at 3 months (*Β* = 0.45, 95% CI = 0.22, 0.68) indicating that for each 1 unit increase in intervention usage score there was an estimated mean 0.45 increase in ASI. The association between intervention usage score and ASI at 6 months was of a similar magnitude (*Β* = 0.48, 95% CI = 0.22, 0.74). The estimated marginal mean ASI scores at 3 and 6 months for different levels of intervention usage are shown in Fig. 1. Exploring the effect of Low, Moderate and High usage scores in the intervention group relative to the control group, the results indicated that at 3 months, the mid usage (M = 57.51, 95% CI = 53.99, 61.04; difference to control M = 5.60, 95% CI = 1.39, 9.81) and high usage groups (M = 60.09, 95% CI = 57.52, 62.67; difference to control M = 8.18, 95% CI = 4.68, 11.68) had significantly higher ASI scores relative to the control group (M = 51.91, 95% CI = 49.58, 54.24). However, these differences were only maintained in the high usage group (M = 60.82, 95% CI = 57.96, 63.67; difference to control M = 8.86, 95% CI = 5.04, 12.68) at 6 months (Table 2; Fig. 2).

**Table 2:** Baseline adjusted ASI-12 by intervention and usage group at 3 and 6 months

	3 Months		6 Months	
	M [95%CI]	Diff. to control M [95%CI]	M [95%CI]	Diff. to control M [95%CI]
Control	51.91 [49.58, 54.24]		51.95 [49.47, 54.44]	
Low usage (0–10.0)	47.49 [41.87, 53.12]	− 4.42 [− 10.55, 1.71]	48.71 [42.47, 54.94]	− 3.25 [− 9.98, 3.49]
Mid usage (10.1–20.0)	57.51 [53.99, 61.04]	5.60 [1.39, 9.81]	55.72 [51.18, 60.27]	3.77 [− 1.40, 8.94]
High usage (20.1–30.0)	60.09 [57.52, 62.67]	8.18 [4.68, 11.68]	60.82 [57.96, 63.67]	8.86 [5.04, 12.68]

Model adjusted for baseline value of the outcome, and study. p-value for group by time interaction = 0.841. There were 34, 60 and 94 participants in the low, mid and high usage groups, respectively

## Discussion

This study examined the association between usage of a m-health intervention app and overall physical activity and sleep health behaviour. In the intervention group there was a weak positive relationship between usage and behaviour at 3 and 6 months. Consistent with this observation, when comparing the Low, Mid and High usage groups to the Control group, only the Mid and High usage groups demonstrated significantly higher (better) overall physical activity and sleep health behaviours at 3 months. These differences were only maintained in the High usage group at 6 months. These results indicate that while there is a weak relationship between intervention usage and behaviour change, it appears that only mid-to-high levels of usage are associated with small improvements in behaviour relative to the control.

Overall, intervention usage scores were modest and there was a weak relationship with behaviour at three and six months. Prior analysis of these trials indicates that overall activity-sleep behaviours did significantly improve in the intervention group relative to the control group [20]. Collectively this suggests that while the use and engagement with the intervention platform has some influence, it is not a major driver of behaviour change. Previous studies have observed no statistically significant [27] or weak [28] associations between various measures of app usage and behaviour change, while others have observed positive dose-response relationships between greater usage and improvements in health outcomes [29]. Similarly, a meta-analysis summarising the association between app engagement and change in physical activity behaviour observed that, while there is a weak statistically significant relationship between usage and physical activity (b = 0.08, 95% CI = 0.01–0.14), there is considerable variation in these associations in different studies [11]. Overall these observations are consistent with conceptual frameworks of engagement-behaviour change that identify platform usage as one of several factors [30, 31], including psycho-social factors related to behaviour change [30], personal relevance of information provided [32], and inclusion of behaviour change techniques in the intervention [31], that can influence behaviour change. This has important implications for the design of future m-health interventions. Specifically, interventions need not only to be designed to promote and foster a certain degree of user engagement with the intervention platform, but they also need to incorporate other important features related to behaviour change. Of relevance to this study which targeted improvements in physical activity and sleep, is the bidirectional relationship between these behaviours [33–35] which may have also influenced behaviour change separate to intervention usage.

The findings from this study suggest that at least a moderate level of usage is required to facilitate greater improvements in ASI scores relative to the Control group (Table 2; Fig. 1). There was no significant difference between the Low usage group and Control group in ASI scores, and the Low usage group had far lower average usage than the moderate and high usage groups. This pattern of results is consistent with suggestions that some level of usage and engagement with digital health interventions is needed to change behaviours [15]. Yet, the optimal amount of intervention usage required to promote behaviour change remains unclear [36, 37], and is likely to depend on an individual characteristics, the outcome targeted and the inherent requirements of the intervention (i.e., daily self-monitoring vs. module based intervention) [37]. Related to this is the issue of intended use relative to actual use of the intervention. While it was intended that participants could self-monitor any of the six physical activity and sleep metrics daily throughout the intervention period, the average usage scores indicate most participants didn’t use the intervention in this way and could be considered non-adherers to the intervention. This relationship is also compounded by the fact that the vast majority of m-health, and digital interventions aim to improve participants’ knowledge and skills to initiate and maintain behaviour change. It is therefore reasonable to assume that at some point, participants stop or reduce their usage because they have acquired the knowledge and skills needed to engage in the behaviour without further use of the intervention, and this declining usage over time, or non-usage attrition, is very common in many e- and m-Health interventions [12, 15, 26, 28, 36].

There are limitations of this study. First, the original trials were powered to detect statistically significant differences in their respective primary outcomes and not to examine the relationship between app usage and behaviour change. Second, the measures of physical activity and sleep used to construct the ASI are self-reported and may be subject to bias. The reporting of sleep quality using the Pittsburgh Sleep Quality Index has demonstrated good reliability [23] and validity in clinical and non-clinical samples [38]. However, although there is some evidence that the Active Australia Survey has acceptable levels of criterion validity, it was designed as a population surveillance instrument and may not be sensitive to detecting changes over time during the intervention [39]. Third, there were some differences between usage groups in terms of baseline behaviour and socio-demographics, which are overcome in part by adjusting statistical analyses for the baseline value of the outcome. Fourth, data on the duration of time spent using the intervention (which has been shown to be associated with behaviour change in other studies [28, 40]) was not captured in the intervention database so could not be examined.

## Conclusion

Overall, there was a weak relationship between app usage and behaviour change in the intervention. Relative to the Control group, only the Mid- and High-usage intervention groups had improved overall patterns of physical activity and sleep behaviours after 3 months, with only the High-usage benefits remaining after 6 months. Collectively these findings suggest that a multidimensional metric of intervention usage has a small influence on behaviour change and that other factors are likely to be key drivers of behaviour change.

## Supplementary information

**Additional file 1: Table S1. **Eligibility criteria, intervention components andoutcome measures used the Synergy and Refresh Studies. **Table S1a.** Operationalisation of social cognitive factorsand behaviour change strategies in the Physical Activity and Sleep HealthIntervention as described in the original Synergy protocol paper. **Table S3** Comparison ofcompleters and non-completers in control and intervention groups pooled fromSynergy and Refresh trials. **Fig. S1**. Screenshots ofBalanced app screens for self-monitoring and feedback relative to goals.

## Data Availability

The datasets used and analysed during the current study are available from the corresponding authors on reasonable request.
